# Field-realistic concentrations of a neonicotinoid insecticide influence socially regulated brood development in a bumblebee

**DOI:** 10.1098/rspb.2022.0253

**Published:** 2022-11-30

**Authors:** Hanna Chole, Miguel de Guinea, S. Hollis Woodard, Guy Bloch

**Affiliations:** ^1^ Department of Ecology, Evolution, and Behavior, Hebrew University of Jerusalem, Jerusalem 91904, Israel; ^2^ Department of Entomology, University of California Riverside, Riverside, CA, USA

**Keywords:** *Bombus terrestris*, neonicotinoid, imidacloprid, body size, brood development, queen behaviour

## Abstract

The systemic neonicotinoid insecticides are considered as one of the key culprits contributing to ongoing declines in pollinator health and abundance. Bumblebees are among the most important pollinators of temperate zone plants, making their susceptibility to neonicotinoid exposure of great concern. We report that bumblebee (*Bombus terrestris*) colonies exposed to field-realistic concentrations of the commonly used neonicotinoid Imidacloprid grew slower, consumed less food, and produced fewer workers, males and gynes, but unexpectedly produced larger workers compared to control colonies. Behavioural observations show that queens in pesticide-treated colonies spend more time inactive and less time caring for the brood. We suggest that the observed effects on brood body size are driven by a decreased queen ability to manipulate the larva developmental programme. These findings reveal an intricate and previously unknown effect of insecticides on the social interactions controlling brood development in social insect colonies. Insecticide influences on the social mechanisms regulating larval development are potentially detrimental for bumblebees, in which body size strongly influences both caste differentiation and the division of labour among workers, two organization principles of insect societies.

## 1. Introduction

Anthropogenic stressors are a key driver of species decline, in part because they negatively affect underlying behavioural and physiological processes that are fundamental to fitness. Pesticides are considered among the most destructive stressors for insects. Their effects, including at sublethal, field-realistic levels, can ultimately reduce fitness through a suite of more subtle effects, many of which cannot be detected by standard practices for assessing pesticide toxicity.

Pollination services provided by diverse animal species contribute substantially to the reproduction of greater than 85% of the flowering vegetal species [1–3]. Bees are important pollinators in both natural and agricultural ecosystems [4–8], yet there is growing evidence that bee populations face severe declines worldwide [9–11]. Multiple anthropogenic stressors have been implicated in the decline of wild and commercially reared bees. These stressors include the intensification of habitat fragmentation and loss [12–15], various pathogens [16–18] and the use of agrochemicals, in particular insecticides [19–21]. The ongoing intensification of agricultural practices implies that bees are expected to experience increasing exposure to agrochemicals. A class of agrochemicals that have received substantial attention is the neonicotinoid insecticides ('neonics'), which are used extensively worldwide [22–24].

Neonicotinoids are neurotoxins that bind to nicotinic acetylcholine receptors (nAChR), leading to paralysis and death of the exposed insect [25–27]. Their systemic form of action allows them to be rapidly absorbed by vegetal tissues and transported to most or all parts of the treated plant, including pollen and nectar in floral tissues [28,29]. Due to their high efficacy, low toxicity to vertebrates and allegedly low environmental contamination, neonicotinoids have become the most widely sold class of insecticides in the world [26,30]. Although their high toxicity enables applying neonics at lower amounts relative to other classes of insecticides, there is a growing body of evidence for their lethal and sublethal effects on non-target insects, including pollinators [31]. These include evidence for negative influences on fertility [32], foraging activity and learning performance [33,34]. This evidence for non-specific toxicity has led to regulatory bans on the use of three commonly used neonic formulations, clothianidin, imidacloprid (IMD) and thiamethoxam, in the European Union and Canada [24,25]. The harmful effects of neonics may vary among species, including among species of bumblebees (*Bombus* spp. [22,35,36]).

Bumblebees are among the most important pollinators in temperate zones, and several species are reared at industrial scales for the purpose of commercial crop pollination, largely in greenhouses [37]. Studies on the effects of neonics on bumblebee biology have revealed a broad spectrum of sublethal negative effects that include reduced food consumption and nest growth [32,38], colony initiation [39,40] and interactions among nestmates [41], and compromised foraging and homing abilities [42–44]. Their canonical effects on the cholinergic system cannot easily explain some of these harmful effects. For example, there is evidence that exposure to neonics resulted in attenuated immune defense [45] and reduced fertility [38,46–48].

Most bumblebee species have an annual life cycle wherein a new colony is founded by a single-mated queen that recently emerged from overwintering [49,50]. Once the first cohort of offspring is produced (which typically develop into worker bees), queens progressively feed them at the larval stage. Later on, when these first worker bees emerge as adults, the colony enters its eusocial stage, which is characterized by overlapping generations of a mother queen and her daughter worker bees, collective care for the brood, and a clear reproductive division of labour [50]. In the presence of workers, queens reduce the time they allocate to activities such as brood feeding, foraging and nest maintenance, and instead focus mostly on reproduction-related activities [51–53]. Workers, in turn, take over most tasks for the nest [54]. The division of labour among workers relates more to body size than to age, with larger worker bees showing morphological and physiological characteristics that appear to make them better fitted for foraging activities ([55–57]; for a recent review see [58]). There is some evidence suggesting that smaller individuals are better in some functions, such as starvation resistance [59,60]. Task performance is flexible in bumblebees, with many workers switching between foraging and nursing activities, even within the same day [57,61]. The queen manipulates offspring development such that queen-reared workers develop over less time, are typically smaller bodied, and are not likely to develop into gynes [52,58]. Accordingly, worker body size is typically smaller at earlier stages of colony development, when queens primarily care for brood, then grow larger along with the emergence of workers that take on brood care activities [53,62].

Here, we explored how exposure to the widely used insecticide Imidacloprid (IMD) influences the dramatic changes in social organization that occur at the early stages of nest initiation in bumblebees. Specifically, we tested the hypothesis that chronic dietary exposure to field-realistic doses of IMD influences brood care behaviour, and thus brood development, in ways that ultimately influence the growth of incipient colonies.

## 2. Methods

### (a) Bumblebee colonies

Fourteen incipient *B. terrestris* colonies were purchased from Polyam Pollination Services (Yad-Mordechai, Israel). Each colony included a queen, 5–10 workers and brood at various stages of development. Upon arrival to our Bee Research Facility (at the E. Safra Campus of the Hebrew University of Jerusalem; Jerusalem, Israel), the entire colonies and wax combs were transferred to fresh wooden nesting boxes (21 cm × 21 cm × 12 cm) equipped with a top, side and front walls made of transparent Plexiglas enabling clear view of the inner parts of the nest. At this stage, the colonies were standardized to only include a queen, four workers and approximately 14 cm^2^ of brood area that included offspring at all stages of development. The four workers were tagged with numbered plastic discs glued to their thoraces (Opalith tags; Christian Graze KG, Germany), allowing for individual identification. Additional information is provided in the electronic supplementary information.

### (b) Experimental set-up

Incipient colonies were randomly assigned to one of three treatments: ‘Control’ treatment, which included five colonies provisioned with sugar syrup with no IMD; the ‘IMD1’ treatment included five colonies fed with sugar syrup in which we dissolved 1 µg l^−1^ of IMD; and the ‘IMD10’ treatment group included four colonies fed with sugar syrup in which we dissolved 10 µg l^−1^ of IMD. Previous studies have shown that these IMD doses are field-realistic (for example: [63–67]). The IMD stock solution (10 mg l^−1^) was produced by diluting 1 mg of IMD (Pestanal, Sigma-Aldrich, USA) solution in distilled water. The stock solution was placed in a glass bottle with an aluminium foil cover to protect from light exposure and was stored in a refrigerator (4°C) until used. Sucrose syrup solutions containing 0, 1.0 and 10.0 µg l^−1^ of IMD were freshly prepared and provisioned by feeders to the experimental colonies every third day throughout the experiment, which lasted a total of 10 consecutive weeks. This duration of exposure mimics field-realistic, long-term exposure to sublethal doses of IMD-treated crops that bumblebee may experience across the foraging season [68,69].

Each focal colony was weighed (to estimate colony growth) every week using a scale (Sartorius Corporation, USA; precision 0.01 g). We used a nest cage, identical to the ones used to house the colonies, installed with an empty plastic feeder (50 ml Falcon tube) and a 50 mm diameter Petri dish to tare the weight of the tested colonies before each measurement. We visually inspected the feeders every third day in order to assess sugar syrup consumption. We used the scale lines (2.5 ml resolution) displayed on their sidewalls for estimating the amount of liquid in each feeder. If the liquid level inside the feeder was approximately in the middle between measurement side-wall lines, the content was estimated as equivalent to the measurement line just below the liquid level plus 1.25 ml (e.g. liquid level in between 40- and 42.5-ml lines—we considered the final content as 41.25 ml). Pollen paste consumption was calculated by subtracting the weight of the Petri dish + pollen pellet at introduction and when removed from the colony 72 h later. Scattered pellets were collected and placed back on the Petri dish feeder before being weighed.

### (c) Assessing worker number and body size of emerging adults

We collected all newly emerging workers, gynes (young queens) and males, in each experimental colony on a near-daily basis (6–7 days a week), individually paint-marked them and estimated their body size, then returned them to their natal colonies. As an index for body size, we used the distance between the two tegulae (wing nod bases; termed below as ‘IT span’) that is highly correlated with other linear body-size measures such as marginal wing cell length and head width [64–66,70–72]. We measured body sizes over the course of 10 consecutive weeks, starting one week after the introduction of the bees to the new cages. We recorded the number of newly emerged workers on each day and used it as an index for colony population growth.

### (d) Behavioural observations

We recorded the behaviour of queens and workers of the Control and IMD10 treatment colonies under dim red light, which bees do not see well [73,74], with minimal disturbance. A single observer recorded the frequency and duration of brood-care activities that included larval feeding, brood incubation, wax pot manipulation and scraping wax from pupal clumps [52,53,57,75]. These tasks were summed together to estimate the total percent of time a focal bee was engaged in brood care. Behaviors such as drinking sugar syrup, eating pollen, strolling in the nest, self-grooming, fanning and inactivity were recorded as non-brood care-related activities [75–77]. The complete list of recorded behaviors and their description is summarized in the electronic supplementary information. We used a focal observation approach in which each observation session included recording queen behaviour for five consecutive minutes, followed by observations of two (individually tagged) workers for 5 min each, sequentially. Focal workers were randomly selected from the bees emerging in the first batch of workers. Observations were performed 3 days a week, over four consecutive weeks, for a total of 60 min per queen and 60 min per worker. All tested colonies were equally observed during morning (between 07:00 and 09:00) or late afternoon (between 16:00 and 18:00) sessions.

### (e) Statistical analyses

Statistical analyses were performed using either R (v. 4.2.1; R Development Core Team 2022) or SPSS (IBM Corp., 2016, v. 24.0, Armonk, NY, USA), with *p* < 0.05 considered statistically significant. The effects of the tested IMD dietary concentrations on colony weight was analysed using the Generalized Estimating Equations test (G.E.E.) with gamma distribution (using SPSS). A first-order autoregressive correlation structure was also considered to account for a serial correlation across the repeated measures. For predictors within GEEs with statistically significant effect, we used pairwise *post hoc* Bonferroni tests. The influence of treatment on the total number of workers, gynes and males per colony was compared using the non-parametric Kruskal-Wallis test.

We used generalized linear mixed models (hereafter ‘GLMMs’) to test the effects of IMD dietary concentrations on colony sugar syrup and pollen consumption, the body size of emerging bees, and queen and worker behaviour (using the *lmer* function from the package ‘lme4’ v. 1.1–30). First, we fitted four GLMMs with a Gaussian error structure and logit link function using syrup consumption (mL) and pollen consumption (g), and worker and gyne body size as response variables. For the models, we used IMD dietary concentration, colony age and their interaction as predictors, and we selected colony as random effect in order to control for the effect of repeated measures per colony. For the GLMM examining difference in gyne body size, we did not include colony age as a predictor variable because gynes emerge only toward the end of colony cycle. To test for the influence of IMD on queen and worker behaviour, we fitted four GLMMs analyses using total time inactive for queens, brood care events of queens, and brood care events of worker bees as response variables. We followed the same structure as above (i.e. same predictors, interactions and random effects) for consistency.

We controlled for type I errors by including all predictors and their interactions as random slopes. We used the ‘car’ package (v. 3.1-0) to assess multicollinearity in our multiple regression models assuming a threshold of 3 for variance inflation factors. We also visually verified that the residuals of our models were normally and homogeneously distributed by plotting them against fitted values as normal quantile and histogram plots. No model assumptions were violated. We compared full and null models including only random effects and offsets using likelihood ratio tests (*anova* function set to ‘Chisq’). If the full model differed from the null model, we next tested which predictors account for the difference. To meet this goal, we used likelihood-ratio tests comparing the full model to reduced models in which one of the predictors was removed from the full model using *drop1* function. For predictors with a statistically significant effect we performed a *post hoc* analyses using Tukey's HSD *posthoc* tests from the package ‘emmeans’ (version 1.8.1-1). Plots show either mean ± standard deviation (s.d.), mean ± standard error (S.E) or boxplots.

## 3. Results

### (a) Influence of IMD on food consumption and colony growth

Chronic oral treatment with IMD affected colony growth and food consumption. At the end of the experiment (day 91), control colonies that were not exposed to IMD weighed on average 533.31 grams, which was 5.65% and 4.59% higher than colonies treated with IMD 1 µg l^−1^ (503.17 ± 50.05 s.d.) and 10 µg l^−1^ (508.84 ± 62.04), respectively. The treatment effect was statistically significant *χ*^2^ (Wald *χ*^2^: 11.756, d.f. = 2, *p* = 0.003; see Methods for details). All colonies increased in weight over time, producing a statistically significant effect of colony age in our analysis (Wald chi-square: 8089.87, d.f. = 11, *p* < 0.001; electronic supplementary material, table S1 and figure S1). The interaction between colony age and IMD treatment was also statistically significant (*p* < 0.001). Pairwise *post hoc* Bonferroni tests revealed significant differences between the Control and the IMD1 group (95% Wald confidence interval for difference: 3.729–52.021, d.f.: 1, *p* = 0.017), and between the Control and IMD10 groups (95% W.C.I.: 5.810 −40.256, *p* = 0.004), but not between the two IMD treatment groups (*p* > 0.999).

The full model for sugar syrup consumption differed from the null model (likelihood-ratio test: *χ*^2^ = 63.65; d.f. = 5; *p*-value < 0.001). The treatment effect was statistically significant (Est.: −5.67 ± s.e. 1.72; CI: −9.04 to −2.30; *p*-value = 0.01), with control colonies consuming more (mean 21.63 ± s.d. 11.35 ml) than colonies fed with either sugar syrup treated with 1 µg l^−1^ (IMD1, mean 16.43 ± s.d. 10.48 ml; pairwise Tukey's HSD test, *t*_52_ = 6.18, *p*-value = 0.008) or 10 µg l^−1^ IMD (IMD10, mean 18.75 ± s.d. 8.95 ml; *t*_52_ = 3.31, *p*-value = 0.074; electronic supplementary material, figure S1). Sugar syrup consumption increased with colony age (Est.: 0.35 ± s.e. 0.03; CI: 0.28–0.34; *p*-value < 0.001) with a steeper increase in control colonies compared to colonies treated with either 1 µg l^−1^ and a similar trend compared to colonies treated with 10 µg l^−1^ IMD (Est.: −0.08 ± s.e. 0.03; CI: −0.15 to −0.02; *p*-value = 0.03; pairwise Tukey HSD test, control versus IMD1: *t*_52_ = 6.18, *p*-value = 0.008; control versus IMD10: *t*(16) = 3.31, *p*-value = 0.007; IMD1 versus IMD10: *t*_35_ = −2.86, *p*-value = 0.22).

The full model differed from the null model also for pollen consumption (likelihood-ratio test: *χ*^2^ = 41.91; d.f. = 5; *p*-value < 0.001). The effect of IMD treatment was statistically significant (Est.: −2.08 ± SE 0.78; CI: −3.62 to −0.53; *p*-value = 0.05) with control colonies (13.31 ± 10.34 g) consuming more than colonies treated with 10 µg l^−1^ (9.14 ± 5.85 g; Tukey's HSD pairwise comparison test; *t*_9_ = 4.48, *p*-value = 0.04) but not 1 µg l^−1^ IMD (9.79 ± 6.71 g; *t*_9_ = 4.35, *p*-value = 0.14), with no difference between colonies exposed to the two IMD concentrations (*t*_8_ = 0.12, *p*-value = 0.79; electronic supplementary material, figure S1). Pollen consumption increased with colony age overall (Est.: 0.24 ± SE 0.03; CI: 0.18–0.29; *p*-value < 0.001), with no significant interaction (Est.: −0.009 ± SE 0.005; CI: −0.019–0.002; *p*-value = 0.28).

### (b) Number of adult bees per colony

**Figure 1.**
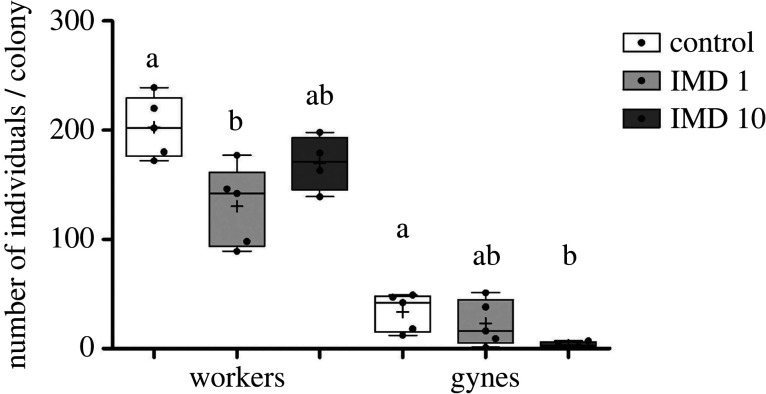
Influence of chronic IMD treatment on the total number of adult bees emerging in a colony. Boxplots with different letters indicating a statistically significant difference in a Kruskal–Wallis (*p* < 0.05) and a Dunn *post hoc* test (*p* < 0.05), which were performed separately for each caste.

IMD-treated colonies produced overall fewer worker bees: IMD1: *x̄* = 130.40, s.d. = 36.44; IMD10: *x̄* = 169.20, s.d. = 44.17; control colonies *x̄* = 202.6, s.d. = 27.70 (figure 1; Kruskal–Wallis; *H* = 7.426, d.f. = 2, *p* = 0.02). Complementary pairwise comparisons demonstrated a significant difference only between the Control and the IMD1 treatment (Dunn post hoc test; *p* < 0.05). Control colonies produced 39.6 gynes (12.46 SD) compared to 10.6 (6.73 SD) and 3.25 (2.62 SD), in colonies treated with 1 and 10 µg l^−1^ IMD, respectively (figure 1, middle panel; *H* = 9.853, d.f. = 2, *p* < 0.01). The difference between the Control and the two IMD 10 treatment was statistically different in complementary pairwise comparisons (*p* < 0.01). The influence of treatment on male production was not statistically significant (*H* = 4.843, d.f. = 2, *p* = 0.089).

### (c) Influence of IMD on offspring body size

**Figure 2.**
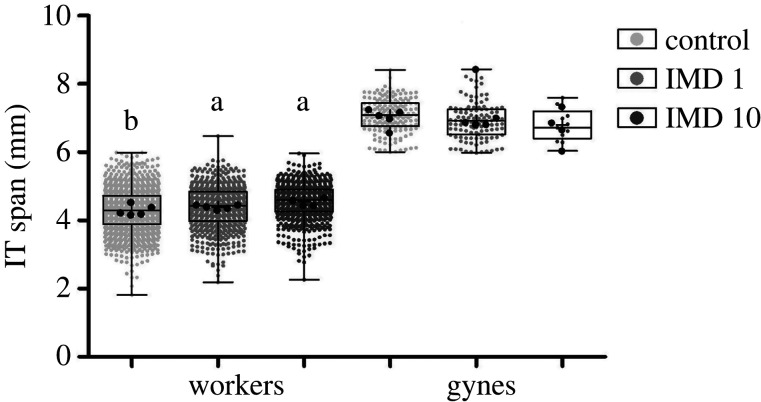
The Influence of chronic IMD consumption on the body size of emerging adult bumblebees. Boxplots with the light small symbols show data of individual bees and larger dark circles show the colony averages for workers gynes and drones. Control: colonies fed with sugar syrup with no imidacloprid; IMD 1: colonies fed with sugar syrup treated with 1 µg l^−1^ IMD; IMD 10: colonies fed with sugar syrup treated with 10 µg l^−1^ IMD. See text for details of statistical analysis.

The full model for worker body size differed from the null model (likelihood-ratio test: *χ*^2^ = 83.115; d.f. = 17; *p*-value < 0.001) with a significant effect for IMD treatment (figure 2; Est.: 1.07 ± s.e. 0.11; CI: 0.863–1.282; *p*-value < 0.001), colony age (Est.: 0.16 ± SE 0.02; CI: 0.123–0.199; *p*-value < 0.001), and a significant interaction between colony age and treatment (Est.: −0.223 ± s.e. 0.02; CI: −0.263 to −0.184; *p*-value < 0.001; electronic supplementary material, figure S2). Worker bees emerging in control colonies were smaller (4.28 ± 0.63 mm) than bees belonging to colonies treated with 1 ug l^−1^ (4.39 ± 0.62 mm; Tukey's HSD test; *t*_12_ = −0.45, *p*-value = 0.006) or 10 ug l^−1^ IMD (4.55 ± 0.52 mm; Tukey's HSD test; *t*_5_ = −0.42, *p*-value = 0.015; figure 2, electronic supplementary material, figure S2). The influence of colony age on worker body size was stronger for bees in control colonies in comparison to worker bees in colonies treated with 1 ug l^−1^ (Tukey's HSD test; *t*_8_ = −0.07, *p*-value = 0.05) or with 10 ug l^−1^ IMD (Tukey's HSD test; *t*_7_ = −0.22, *p*-value = 0.04). Contrary, the comparison between full and null models testing for differences in gynes size was not statistically significant (likelihood-ratio test: *χ*^2^ = 1.305; d.f. = 2; *p*-value = 0.521), but this could be due to the small number of gynes emerging in IMD10 colonies (figure 2).

### (d) Brood-care activities

**Figure 3.**
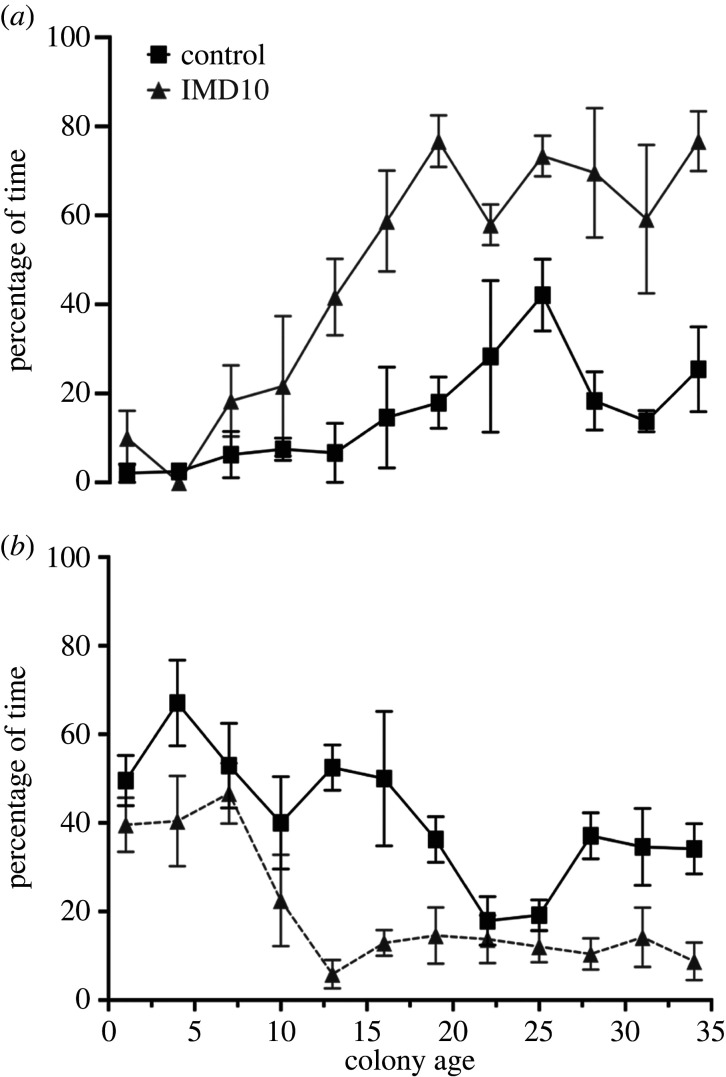
Influence of IMD on queen activity and brood care behaviour. (*a*) Percentage of time the queen was recorded inactive (motionless). (*b*) Percentage of time the queen spent performing brood care-related activities. The queens were observed over 5 min sessions that were performed three times a week, over five consecutive weeks. Data show mean and SE.

Queens in the IMD10 treated colonies were recorded motionless more often (likelihood-ratio test:^2^ = 25.56, d.f. = 5, *p*-value < 0.001; Est.: 61.22 ± SE 17.75, CI: −3.62 to −0.53, *p*-value = 0.03; figure 3*a*). There was also an effect of colony age with the queens of older colonies spending more time inactive (colony age; Est.: 13.81 ± SE 2.48, CI: 8.96–18.64, *p*-value = 0.001). The slope of increased inactivity over time was higher for queens in colonies treated with 10ug/l IMD in comparison to control colonies (Est.: 13.28 ± SE 3.43, CI: 6.56–19.98, *p*-value < 0.001). The IMD10 queens performed fewer brood care-related tasks (likelihood-ratio test: *χ*^2^ = 23.73, d.f. = 5, *p*-value < 0.001; Est.: −62.58 ± s.e. 10.04, CI: −82.26 to −42.91, *p*-value = 0.03; figure 3*b*) compared to the queens of control colonies. Queen brood care behaviour overall decreased with colony age (Est.: −8.65 ± SE 1.49, CI: −11.59 to −5.73, *p*-value < 0.001) but we did not find a significant interaction between treatment and colony age (Est.: −0.64 ± s.e. 3.13, CI: −6.78–5.49, *p*-value = 0.87).

Worker bees also showed lower levels of brood care-related behaviours in IMD-treated colonies (likelihood-ratio test: *χ*^2^ = 20.74, d.f. = 3, *p*-value < 0.001; Est.: −14.86 ± s.e. 5.96, CI: −26.54 to −3.88, *p*-value = 0.02). Worker brood care-related behaviour significantly decreased with colony age (Est.: −8.09 ± s.e. 2.15, CI: −12.30 to −3.88, *p*-value < 0.001) with a steeper decrease in control colonies than in colonies treated with 10 ug/L IMD (Est.: 9.33 ± s.e. 3.89, CI: 1.71–16.94, *p*-value = 0.018), probably because of the rapid increase in the number of brood tending workers during the logarithmic phase of colony growth.

## 4. Discussion

Here, we reveal that exposure to sublethal levels of a neonicotinoid insecticide changes brood care behaviour of bumblebee queens, and the size distribution of offspring in young nests. Specifically (and counterintuitively), pesticide exposure led to the production of larger workers, but not larger gynes (which actually showed a trend towards smaller size in IMD-treated colonies). Our observations of worker and queen behaviour may help explain the production of larger-bodied workers in pesticide-treated nests. Queens in our insecticide-treated colonies exhibited decreased activity and reduced brood tending. Bumblebee queens have been shown to rear smaller-bodied offspring, relative to offspring reared by workers (e.g. *B. terrestris* [53]; *B. impatiens* [62]). Thus, a shift toward more worker brood care (relative to queen brood care) in the IMD-treated nests might have caused a shift toward the production of workers with a larger body size, which is determined during the larval stage in bumblebees [53,58,62]. Overall, these findings show complex influences of field-realistic, sublethal doses of a commonly used insecticide, operating specifically on the social biology of an ecologically and economically important pollinator species.

Given that the IMD-treated colonies consumed less food and produced fewer workers (which can bring food to larvae), it was unexpected to find that the workers emerging in these colonies were larger than in our control colonies. The body size of adult bumblebees is strongly influenced by the amount of food provisioned at the larval stage, and the overall quality of their care by tending adults [42,62,78–80]. Although we did not assess queen fecundity directly, negative effects of the pesticide on queen fertility (reviewed by Camp & Lehmann [81]; see also [39,40,47,82]) may have led to a reduced number of offspring in the colonies. Brood care and other relevant factors, such as colony temperature and maintenance, are influenced by many factors including interactions between the queen, workers, and the developing brood [41,58,83]. Insecticides that modify adult bee behaviour or social interactions (such as imidacloprid [39–42]) may therefore lead directly or indirectly to changes in aspects of brood care that ultimately influence offspring size. For example, both IMD and the widely used herbicide Glyphosate impair bumblebee ability to thermoregulate their colonies, which can have a significant effect on the body size of emerging bees [41,83].

Queens of IMD-fed colonies were more commonly observed motionless (an effect also observed in *B. impatiens* [39]) and were specifically observed tending brood less often (figure 3b). Worker brood-tending activity decreased over time, with a sharper decrease in control colonies and lower levels during the first few weeks. This decrease over time may be explained by the increase in worker number, allowing the two focal workers to provide less care. In *B. terrestris,* larvae that are tended by queens develop faster, are commonly smaller, and are unlikely to develop into gynes [53,84]. These differences between the development of larvae tended by the queen or the workers support a self-organized model explaining the typical gradual increase in worker size along with colony growth and the switch from worker to gyne production toward the end of colony life [53,58]. The increase in the number of worker bees further allows the queen to reduce brood tending and increase egg-laying activity, which may further increase colony population size, and dilute her influence on larval development [51,52]. Given that the queens of IMD-treated colonies provide less care for the larvae, which we suggest could escape at least part of her effects, ultimately developing into larger adults in the ad libitum fed laboratory colonies used for this study.

Our finding of larger-bodied workers in IMD-treated colonies differs from a field study in which *B. terrestris* colonies that were placed in clothianidin-treated oilseed rape fields had smaller workers and males [80]. Although we cannot exclude the possibility that these differences relate to the use of two different neonics, we believe that a likely explanation relates to differences between laboratory and field experiments. It is also worth noting that bumblebees in our study were only exposed to imidacloprid through sugar syrup, whereas in the field they may in addition be exposed to IMD in the pollen. Neonics influence various neurological functions such as orientation, learning and memory and circadian rhythms that influence foraging performance [85,86]. Thus, field colonies exposed to neonics may overall have a lower pollen and nectar supply, higher mortality leading to under-nourishment, and various compensation mechanisms that may increase metabolic costs and affect colony thermoregulation and overall homeostasis. There are also reports suggesting a general trend toward colonies producing smaller individuals in habitats subjected to anthropogenic influences [87,88]. Thus, although the effects on the queen may be similar in the field to what we found in our laboratory study, the undernourished field colonies cannot provision sufficient food to the larvae they rear, which therefore develop into smaller individuals than in neonic-free colonies. On the other hand, laboratory colonies invest significantly less energy in food gathering and processing (i.e. all workers are involved in in-nest activities), have an ad libitum food supply, and live in a constantly regulated environment, requiring them to devote less energy and time for regulating temperature and other aspects of the colony microenvironment (for example, see [41,83]). Additional studies in the laboratory and field are important for allowing us to uncouple foraging or colony homeostasis-related effects from influences on behaviour, physiology and social interactions inside the nest.

Our findings corroborate the multifaceted effects of neonicotinoids on pollinator health and function and add to the small but growing cannon of studies showing that in social insects, these insecticides can have more nuanced, disruptive effects on intricate social processes. These include impacts on social interactions between adults and developing brood [41,83] that are essential for colony growth and fitness. We suggest that our observed effects on offspring body size are at least partially explained by IMD effects on queen behaviour specifically, which reduces her capacity to contact young larvae and manipulate their developmental programme [53,62]. The seemingly opposite effects of IMD on gyne body sizes may be related to the fact that gynes are produced late in the season when the colonies are larger and queens have ceased providing brood care, and thus the queen has little effect, if any at all, on larval development. The effects we observed should be further tested, given that our study is based on a limited number of colonies per group, and the results might be different under alternative food resource conditions, which can reveal novel pesticide effects [62,83]. Insecticide effects on larval development and ultimate body size may be specifically significant for bumblebees because body size is intricately connected with both caste determination and the division of labour among workers in this bee lineage. Thus, factors that influence female body size may hamper task performance, task allocation and overall colony performance [57,58,62,76,89,90]. Moreover, there is evidence that the body size of both gynes and males may affect their mating and reproductive success [72,91–93]. Thus, insecticides with sublethal effects on the regulation of brood development may have an unexpectedly significant influence on colony fitness.

## Data Availability

All our data is provided in the paper or as electronic supplementary material [94].
